# Distinct Morphology of Human T-Cell Leukemia Virus Type 1-Like Particles

**DOI:** 10.3390/v8050132

**Published:** 2016-05-11

**Authors:** José O. Maldonado, Sheng Cao, Wei Zhang, Louis M. Mansky

**Affiliations:** 1 Institute for Molecular Virology & DDS-PhD Dual Degree Program, 18-242 Moos Tower, 515 Delaware Street SE, Minneapolis, MN 55455, USA; jmaldo@umn.edu; 2 Institute for Molecular Virology, 18-242 Moos Tower, 515 Delaware Street SE, Minneapolis, MN 55455, USA; caosheng@wh.iov.cn; 3 Institute for Molecular Virology & Characterization Facility, 18-242 Moos Tower, 515 Delaware Street SE, Minneapolis, MN 55455, USA

**Keywords:** deltaretrovirus, lentivirus, virus assembly

## Abstract

The Gag polyprotein is the main retroviral structural protein and is essential for the assembly and release of virus particles. In this study, we have analyzed the morphology and Gag stoichiometry of human T-cell leukemia virus type 1 (HTLV-1)-like particles and authentic, mature HTLV-1 particles by using cryogenic transmission electron microscopy (cryo-TEM) and scanning transmission electron microscopy (STEM). HTLV-1-like particles mimicked the morphology of immature authentic HTLV-1 virions. Importantly, we have observed for the first time that the morphology of these virus-like particles (VLPs) has the unique local feature of a flat Gag lattice that does not follow the curvature of the viral membrane, resulting in an enlarged distance between the Gag lattice and the viral membrane. Other morphological features that have been previously observed with other retroviruses include: (1) a Gag lattice with multiple discontinuities; (2) membrane regions associated with the Gag lattice that exhibited a string of bead-like densities at the inner leaflet; and (3) an arrangement of the Gag lattice resembling a railroad track. Measurement of the average size and mass of VLPs and authentic HTLV-1 particles suggested a consistent range of size and Gag copy numbers in these two groups of particles. The unique local flat Gag lattice morphological feature observed suggests that HTLV-1 Gag could be arranged in a lattice structure that is distinct from that of other retroviruses characterized to date.

## 1. Introduction

Approximately 10–20 million people are infected with human T-cell leukemia virus type 1 (HTLV-1) worldwide [1,2]. HTLV-1 is a deltaretrovirus and is associated with adult T-cell leukemia/lymphoma, tropical spastic paraparesis, as well as HTLV-1-associated myelopathy [3,4]. These diseases are prevalent in places highly endemic for HTLV-1 infection such as southwestern Japan, central Africa, South America and the Caribbean. Despite the association of HTLV-1 with cancer and its significant impact on human health and well-being, the molecular mechanisms of viral replication, virus particle assembly and morphology remain poorly understood due to difficulties in propagating the virus in tissue culture.

Like other retroviruses, the assembly and budding of HTLV-1 particles is directed by the viral Gag polyprotein (recently reviewed by Maldonado *et al.* [5]). Briefly, HTLV-1 Gag molecules translocate to the plasma membrane (PM) soon after the protein is synthesized [6]. A previous study with human immunodeficiency virus type 1 (HIV-1) suggested that the viral RNA is recruited to the PM by Gag and serves as a platform to promote Gag-Gag interactions, allowing Gag to form higher order oligomers in immature particles [7]. Infectious virions are produced via a maturation process that occurs either concomitantly with or after budding of the immature virus. During virus maturation, the viral protease (PR) cleaves the Gag polyprotein into three structural proteins: matrix (MA), which remains associated with the inner leaflet of the viral membrane; capsid (CA), which organizes into a closed protein shell to package the genomic RNA; and nucleocapsid (NC) which is in complex with the viral genome.

The diameters of retrovirus particles are typically variable and commonly appear to form a normal distribution [8,9,10,11,12,13,14,15,16]. Calculations of the average Gag copy number per virus particle vary somewhat depending on the methods used for the measurement as well as on the type of retrovirus being analyzed. Scanning transmission electron microscopy (STEM) has previously been used successfully to determine the average Gag copy number per particle [9,10,15,16,17,18]. This method estimates the mass of the whole virus particle. Since the majority of the virus particle mass is contributed by Gag, the mass of the entire particle has been used for calculating the Gag stoichiometry. Multiple studies have reported varying Gag copy numbers, ranging from approximately 750 to 5000, which coincide with varying virus particle size distributions [10,12,13,15,16,17,18,19]. To date, there are no reported studies on Gag stoichiometry that would be present in the immature precursors of authentic, mature HTLV-1 particles. Determination of Gag stoichiometry is critical to understanding the mechanisms of HTLV-1 replication, for this information assists in the interpretation of HTLV-1 particle structures, and helps in determining the copy number of other viral proteins in the virus particle (e.g., Pol).

In this study, a comparative analysis of HTLV-1-like particles and authentic, mature HTLV-1 particles was performed by cryogenic transmission electron microscopy (cryo-TEM) and scanning transmission electron microscopy (STEM). These findings provide the first demonstration of the morphology of these virus-like particles (VLPs) having the unique feature of local flat Gag lattice regions that did not follow the curvature of the viral membrane and had an enlarged distance toward the membrane. Morphological features similar to that observed with other retroviruses [20] include (1) a Gag lattice with multiple discontinuities; (2) a string of bead-like densities at the inner leaflet that is associated with the Gag lattice; and (3) a Gag lattice resembling a railroad track. We also demonstrate that HTLV-1-like particles and authentic mature HTLV-1 particles possess a consistent size and Gag stoichiometry.

## 2. Materials and Methods

### 2.1. Transfection and HTLV-1-Like Particle Production

**Figure 1:**
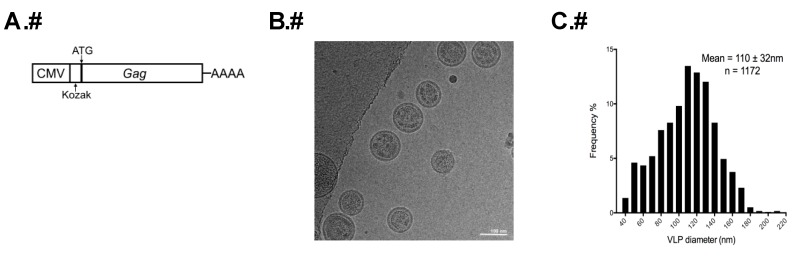
Analysis of the diameter and morphology of human T-cell leukemia virus type 1 (HTLV-1) virus-like particles (VLPs) by transmission electron microscopy (TEM). (**A**) HTLV-1-like particle expression construct. A codon-optimized Gag expression construct (pN3 HTLV-1 Gag) with a Kozak sequence was used to produce HTLV-1 VLPs; (**B**) Representative micrograph of HTLV-1-like particles of different sizes and morphology; (**C**) Size distribution of HTLV-1-like VLPs.

A codon-optimized HTLV-1 *gag* gene expression construct (pN3 HTLV-1 Gag, Figure 1A) was created in a similar manner to that of a previously described construct in which the yellow fluorescence protein (YFP) was fused to the carboxy-terminus of Gag (pEYFP-N3 HTLV-1 Gag) [21]. The new *gag* gene which does not have a YFP tag was synthesized with an optimal Kozak consensus sequence at the 5′ end of the gene: GCCACC**ATG**G (start codon in bold and underlined) (Figure 1A). In order to produce VLPs, six 10 cm tissue culture dishes each containing 2.2 × 10^6^ human embryonic kidney 293T cells in 6 mL of Dulbecco's Modified Eagle Medium (DMEM) supplemented with 10% Fetal Clone III were co-transfected with the pN3-HTLV-1 Gag expression construct along with an HTLV-1 envelope protein expression construct (ratio of 10:1) using GeneJet (SignaGen, Gaithersburg, MD, USA) following the manufacturer’s instructions. Twenty-four hours post-transfection, 2 mL of fresh media were added to each plate and incubated for additional 24 h at 37 °C in 5% CO_2_. To harvest VLPs, cell culture supernatants from transfected cells were centrifuged at 3000× *g* for 5 min to remove large cellular debris and then filtered through a 0.2 µm filter. The samples were then concentrated and purified in the same manner as with authentic particles.

### 2.2. Gradient Purification of Authentic Virus Particles and VLPs

Authentic HTLV-1 particles were produced from MT-2 cells, a T-cell line chronically infected with HTLV-1, which was obtained from Dr. Douglas Richman through the NIH AIDS Reagent Program, Division of Acquired Immune Deficiency Syndrome (AIDS), National Institute of Allergy and Infectious Diseases (NIAID), National Institutes of Health (NIH) [22,23]. MT-2 cells were grown in two T-75 flasks with up to 60 mL of Roswell Park Memorial Institute (RPMI) 1640 medium supplemented with 10% Fetal Clone III, for ~10 days. After the cells reached about 90% confluency, which was indicative by the formation of large cell clumps, virus particles were harvested and centrifuged at 3000× *g* for 5 min to remove large cellular debris and then filtered through a 0.2 µm filter.

The concentrated particles (*i.e.*, authentic virus particles or VLPs) were then ultracentrifuged through an 8% OptiPrep (60% iodixanol in water with a density of 1.32 g/mL, (Sigma-Aldrich, St. Louis, MO, USA) cushion at 109,000× *g* for 1.5 h in a 50.1 Ti rotor (Beckman, Brea, CA, USA) at 4 °C. The particle pellet was resuspended in 0.5 mL of 1× STE buffer (100 mM NaCl , 10 mM Tris-Cl, pH 7.4, 1 mM sodium chloride-Tris-ethylenediaminetetraacetic acid (EDTA), and overlaid onto a 4 mL 10%–40% OptiPrep gradient and centrifuged to equilibrium in a SW55 Ti rotor (Beckman) at 250,000× *g* for 3 h at 4 °C. The virus- or VLP-containing fraction, at about 20% OptiPrep, was removed from the gradient using a hypodermic needle. The collected virus particles were diluted 10 fold in 1 × STE and pelleted at 195,000× *g* for 1 h in a SW55 Ti rotor at 4 °C. Following centrifugation, the pellet was re-suspended in ~15 µL of 1 × STE at 4 °C overnight and then analyzed by cryo-TEM or STEM. The compound 2, 2′-dithiodipyridine (aldrithiol-2; AT-2) was used to inactivate authentic HTLV-1 infectivity prior to cryo-TEM or STEM analysis as previously described [24].

### 2.3. Cryo-TEM of HTLV-1-Like Particles and Authentic Virus Particles

Virus and VLP samples were prepared for cryo-TEM as previously described [21]. Briefly, 3 µL concentrated virus or VLP sample was applied to a glow-discharged c-flat holey carbon grid (Ted Pella, Redding, CA, USA) and then blotted with filter paper to remove the sample excess. The grid was then plunged frozen into liquid ethane [25] with a FEI MarkIII Vitrobot system (FEI Company, Hillsboro, OR, USA). The frozen grids were then transferred to a FEI TF30 field emission gun transmission electron microscope at liquid nitrogen temperature (FEI Company). Images were then recorded at a nominal magnification of 39,000_x and 59,000_x at low-dose (~30 electrons/Å^2^) and 1 to 5 µm under focus conditions using a Gatan 4 k by 4 k CCD camera (Gatan Inc., Pleasanton, CA, USA).

### 2.4. Determination of Particle Size

Cryo-TEM images were analyzed by using ImageJ software (Version 1.49c, NIH, Bethesda, MD, USA). For each virus particle or VLP analyzed, two perpendicular diameters were used to calculate the average diameter [21]. Histograms of particle diameters were generated by using GraphPad Prism 6 software (Version 6.0c, GraphPad, La Jolla, CA, USA).

### 2.5. Determination of Particle Mass by STEM

The mass of virus particles or VLPs was determined by quantitative dark-field STEM, which was developed at the Brookhaven National Laboratory (BNL, Upton, NY, USA) [26]. This method allows for the study of individual unstained virus particles with minimal radiation damage. The particle sample was first mixed with tobacco mosaic virus (TMV) particles that were used as an internal control. Then the mixture was applied onto a thin-carbon transmission electronic microscopy (TEM) grid, extensively washed, blotted and freeze-dried overnight. The TEM grid was imaged under a 40 keV electron beam at −150 °C. The grid was first scanned with a low dose electron beam and areas with clean background were used for the final scan. Prior to the final scanning, the electron beam was focused in a nearby area to minimize radiation damage to the specimen. The low temperature and low dose imaging technique (<500 electrons/nm^2^) was used to help to reduce mass loss (less than 1%) caused by electron radiation as well as to eliminate contamination from mobile hydrocarbons. Each point in the STEM image corresponds to an area of 0.625 nm^2^ over the specimen. The whole image corresponds to 512 by 512 nm in the specimen with the center of the points separated by 1 nm. A large- and a small-angle annular dark-field detector were used to digitally record the number of scattered electrons in each scanning point. The number of scattered electrons at any scanning point is proportional to the sample mass in that local region.

STEM images were analyzed by using the PCMass software developed by the BNL STEM facility (Version 32, Brookhaven National Laboratory, Upton, New York, USA). Each virus particle or VLP in the STEM micrograph was first masked by a density profile model (*i.e.*, a sphere) in order to mimic the virus density profile. The diameter of the sphere was based on the dimension of the measured particle. The mass of each virus particle was then calculated using the sum of the electron densities within the mask and a scale factor, which was determined using the image of TMV and its mass per unit length (*i.e.*, 13.1 kDa/Å) [26,27]. The resulting histograms and graphs of particle mass distribution were generated by using GraphPad Prism 6 software (Version 6.0c, GraphPad, La Jolla, CA, USA).

## 3. Results

### 3.1. Analysis of the Morphology of HTLV-1-Like Particles

**Figure 2:**
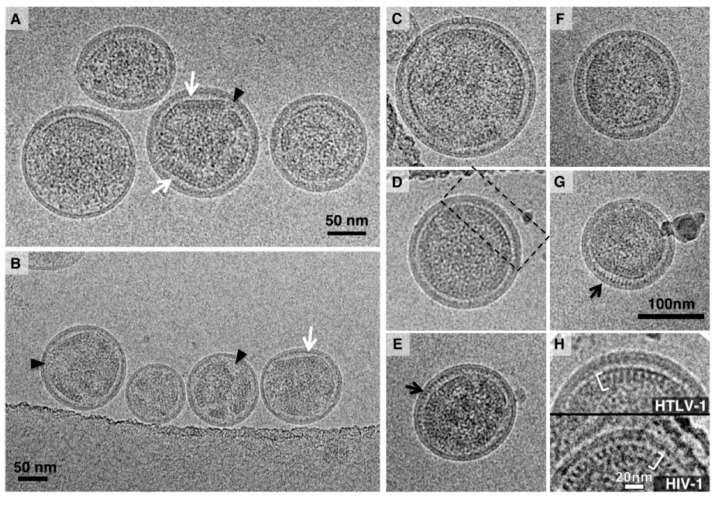
Cryogenic transmission electron microscopy (Cryo-TEM) images of HTLV-1-like particles and comparison of Gag lattice between HTLV-1 and human immunodeficiency virus type 1 (HIV-1). (**A–G**) Cryo-TEM images of HTLV-1-like particles. The white arrows indicate regions of the Gag lattice that appear flat in contrast to the curvature observed with the viral membrane. The black arrows show the membrane regions that are associated with Gag lattice and exhibit a string of bead-like densities in the inner membrane leaflet. The black arrowheads demark discontinuity of the Gag lattice. The black dash-lined box in D shows a region displayed in the top panel of H. The scale bar in G is applicable to the panels C–G; (**H**) Comparison of Gag lattice morphology between HTLV-1-like and HIV-1-like particles. The electron densities representing the Gag lattice structure are indicated by the left and right bracket, respectively.

HTLV-1-like particles produced using the HTLV-1 Gag-only expression construct (Figure 1A) were observed to be spherical in shape with a mean diameter of 110 ± 32 nm measured from 1172 particles (Figure 1B,C). This is in contrast to a previous study using a Gag-YFP expression construct in which a mean particle diameter of 71 ± 20 nm was determined by cryo-TEM [21]. The electron density adjacent to the inner viral membrane was interpreted as being the immature Gag lattice. All particles with this electron density pattern were counted as Gag-containing particles. Intriguingly, many local regions of Gag assembly were observed to exhibit flat electron density features that did not strictly follow the curvature of the membrane and showed enlarged distance toward the viral membrane (4 *vs.* 8 nm) (Figure 2A,B). About 20% of particles had this morphological feature. This unique structural feature has not been reported for other retrovirus immature particles. The flat Gag density feature observed with HTLV-1-like particles suggests that HTLV-1 Gag could be arranged in a lattice structure that is distinct from that of other retroviruses characterized to date (*i.e.*, HIV-1, Mason-Pfizer monkey virus (MPMV) and Rous sarcoma virus (RSV)) [13,28,29].

Other morphological features have commonalities with other retroviruses. First, the Gag densities in the cryo-TEM images were not always continuous. In particular, smaller VLPs were observed to have multiple discontinuities in the immature Gag lattice (Figure 2A,B). The membrane regions that associate with organized Gag lattices appear to be wider and more pronounced. Between the Gag lattice and the viral membrane, a string of bead-like densities is sometimes observed (*i.e.*, in ~10% of particles analyzed) lining along the inner leaflet of the viral membrane (Figure 2E,G). These density features are likely due to the association of MA with the inner membrane of the virus particle. The MA lattice has been observed in a large membrane-enclosed multi-core structure in supernatants of HIV-1-infected cells [30]. In the cryo-TEM images (Figure 2A-G), the arrangement of the Gag molecules within the lattice is similar but not identical to that in other retrovirus immature particles such as HIV-1. The cryo-TEM image (Figure 2H) and cut-away view of the HIV-1 Gag lattice assembly in a three-dimensional (3D) reconstruction map [31,32] has two density layers: closer to the viral membrane is the CA protein layer showing an array of rod-like densities, while towards the center of the particle is the NC layer that has a continuous density. In contrast, the HTLV-1-like particles consistently display a continuous density at the region closer to the membrane inner leaflet. Underneath the continuous density layer, closer to the center of the virus particle, is an array of densities that resembled a railroad track (Figure 2H).

### 3.2. Morphology of Authentic HTLV-1 Mature Particles Produced from MT-2 Cells

**Figure 3:**
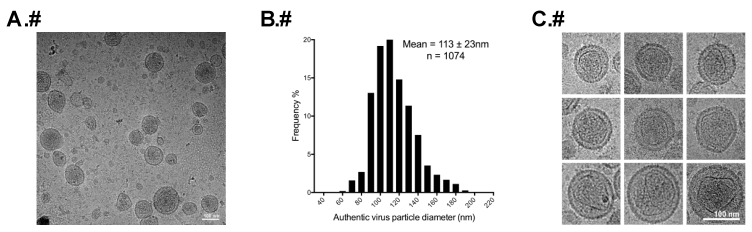
Analysis of the diameter of authentic mature HTLV-1 virus particles. (**A**) Authentic mature HTLV-1 particles produced from MT-2 cells; (**B**) Size distribution of authentic mature HTLV-1 particles; (**C**) Magnified images of authentic mature HTLV-1 particles showing irregular polyhedral-like core structures. The scale bars in A and C are 100 nm.

As a control and to confirm our previous studies with authentic HTLV-1 particles by cryo-electron tomography [33], the morphology of authentic HTLV-1 particles, harvested and purified from MT-2 cells [22,23], was also studied by cryo-TEM (Figure 3A). Mature virus particles were identified by either the presence of readily observable electron-dense cores, or by the presence of significant electron density within the particle. The lack of HTLV-1 protease inhibitors prevented the production of large numbers of authentic immature particles. Vesicles were identified by the absence of core structures or significant internal electron density. The particles were primarily spherical and heterogeneous in size. The particle diameter was determined by averaging the longest and shortest measurements of each particle. A total of 1074 authentic particles were measured and had a mean diameter of 113 ± 23 nm (Figure 3A,B). This measurement based on two-dimensional (2D) cryo-TEM images was in good agreement with our previous analyses using the cryo-electron tomography method [33].

A gallery of cryo-TEM images of authentic HTLV-1 particles (Figure 3C) revealed that the particles contained an unordered polyhedral-like capsid core structure, which is different in each particle regardless of particle size. The core size varied by particle, with some regions of the protein capsid of the cores following the curvature of the inner leaflet of the viral lipid bilayer, while other parts of the capsid appeared completely separated from the viral membrane.

### 3.3. STEM Analyses of HTLV-1-Like Particles and Authentic Mature HTLV-1 Particles

**Figure 4:**
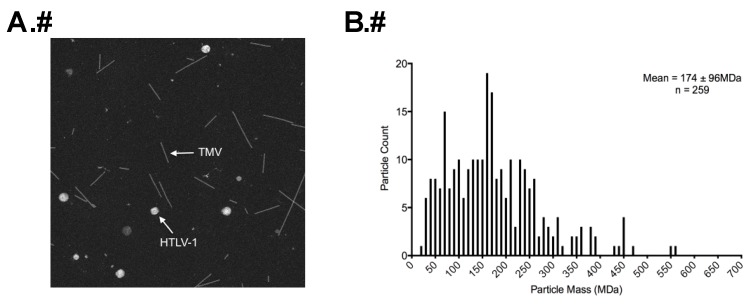
Scanning transmission electron microscopy (STEM) analysis of HTLV-1-like particles. (**A**) A STEM micrograph of HTLV-1-like particles mixed with tobacco mosaic virus (TMV); (**B**) TMV-corrected mass measurement distribution in MDa of purified HTLV-1-like particles, which was determined based on the known TMV mass per unit length of 13.1 kDa/Å.

**Figure 5:**
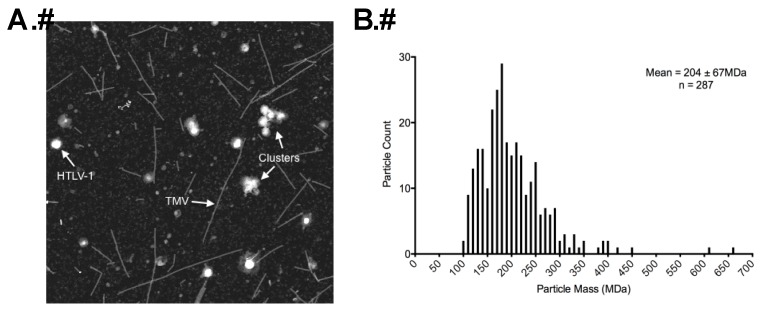
STEM analysis of authentic mature HTLV-1 virus particles. (**A**) A STEM micrograph of authentic HTLV-1 particles mixed with TMV. The region labeled as “clusters” represents closely associated viral particles and is excluded from the calculation; (**B**) The TMV-corrected measurement of mass distribution in MDa of purified authentic mature HTLV-1 particles was determined. The TMV-corrected particle mass determination was based on the known TMV mass per unit length of 13.1 kDa/Å.

STEM analysis was used to determine the total molecular mass of HTLV-1 particles as previously described [26]. Representative dark-field electron micrographs of HTLV-1-like particles and authentic mature HTLV-1 particles are shown in Figure 4A and Figure 5A. Only isolated intact particles that were of the expected particle diameter range, as determined by cryo-TEM imaging, were used for mass measurements. Some smaller randomly distributed contaminants are visible in the background. Using the known mass of TMV as an internal control, we are able to obtain the average masses of HTLV-1 VLPs and authentic particles.

Both HTLV-1-like particles and authentic particles showed a wide distribution of mass diversity, which correlates with the wide particle size distribution (Figure 4B and Figure 5B). The TMV-corrected masses of HTLV-1-like particles (Figure 4B) and HTLV-1 authentic particles (Figure 5B) were determined to be 174 ± 96 MDa and 204 ± 67 MDa, respectively (Table 1). The average masses were used for estimating the Gag copy numbers in HTLV-1-like particles and inferred immature precursors of the authentic HTLV-1 particles. AT-2 was used to inactivate the particles. It is formally possible that AT-2 treatment could affect particle morphology.

### 3.4. Calculation of Gag Stoichiometry in HTLV-1-Like Particles

The average mass of HTLV-1-like particles determined by STEM was used to estimate the average Gag copy number per virus particle. The viral RNA mass contribution from total particle mass was determined by extracting the RNA from particle lysates with RNA columns, using Roche’s High Pure Viral RNA Kit (Roche Diagnostics, Indianapolis, IN, USA). The extracted viral RNA was quantified by determining the ultraviolet (UV) absorption at 260 nm and a conversion factor of 40 µg/mL × A260 optical density unit x dilution factor using a Beckman DU-65 spectrophotometer (Beckman Coulter, Brea, CA, USA). The Thermo Scientific Pierce BCA Protein Assay Kit (Thermo Fisher Scientific, Waltham, MA, USA) was used to estimate the protein content of the same sample used to determine the VLPs’ RNA content. The VLPs’ Gag/RNA mass ratio was determined to be 14.4:1, equivalent to about 4% of the averaged molecular mass of the VLPs measured by STEM (Table 1). Based upon the average size of the VLPs, which is 110 nm, and estimating the average thickness of the viral membrane to be 5 nm, an estimate of the number of lipid molecules in the virus envelope was made. Assuming that the distance between lipid molecules in the same leaflet was 0.85 nm [34], and the average molecular weight of lipids was 750, the mass of lipids in an averaged size particle was determined to be approximately 70 MDa (*i.e.*, ~40% of the total mass of an averaged sized particle) (Table 1).

Assuming that the mass of the Gag protein in HTLV-1-like particles is similar to that of other retroviruses, approximately 70%–90% of the total protein mass [17,35], the mass contribution of Gag in an HTLV-1-like particle with an average size of 110 nm would be 70–87 MDa. Given the molecular weight for HTLV-1 Gag is ~53 kDa [36,37], it was estimated that HTLV-1-like particles contain approximately 1300–1600 Gag polyproteins per VLP with a mass and diameter of 174 MDa and 110 nm, respectively (Table 1).

### 3.5. Estimating Gag Stoichiometry in Authentic Immature HTLV-1 Particles by Calculating Gag Copy Number in Authentic Mature HTLV-1 Particles

The same methodology used to calculate the Gag copy number in HTLV-1-like particles was used to estimate the Gag stoichiometry in authentic immature HTLV-1 particles by calculating the Gag copy number in authentic mature HTLV-1 particles. Based upon the average size of the authentic mature HTLV-1 particles, which is 113 nm, we estimated the lipid mass to be approximately 80 MDa (Table 1). The viral RNA in authentic HTLV-1 particles was calculated by assuming that each authentic particle contains two copies of the 8.5 kb genomic RNA, plus tRNA and other small RNAs comprising approximately 30% of the genomic RNA by mass. The molecular weight of RNA was estimated to be 7 MDa, equivalent to about 3.5% of the averaged molecular mass of the particle measured by STEM. Assuming that the mass of the Gag protein in retroviruses is about 70%–90% of the total protein, the Gag protein of authentic HTLV-1 particles would contribute 82–106 MDa to the total particle mass for a particle of an average size of 113 nm (Table 1). An authentic HTLV-1 particle contains three forms of the Gag polyprotein: Gag, Gag-Pro and Gag-Pro-Pol, with molecular weights of 53 kDa, 76 kDa and 180 kDa, respectively [36,37]. Given that the estimated molar ratio of the Gag, Gag-Pro, and Gag-Pro-Pol is 100:10:1 based on *in vitro* translation of viral RNA [38], it was calculated that the immature precursor of authentic HTLV-1 particles contains approximately 1500 to 1900 copies of Gag, which would result in a particle with a mass of 204 MDa and a size of 113 nm (Table 1) [34,38].

## 4. Discussion

Although HTLV-1 was the first human retrovirus to be discovered [39,40], the morphological details of HTLV-1 particles have been poorly characterized, including that of Gag stoichiometry. To combat the technical difficulties in working with HTLV-1 in cell culture, a HTLV-1 Gag-only expression model system was used to produce and purify HTLV-1-like particles. A key technical advantage of this HTLV-1 Gag model system is that it is a highly robust system that results in highly efficient production of VLPs from mammalian cells. In the absence of methodologies to efficiently produce authentic immature HTLV-1 particles, and given the absence of HTLV-1 PR inhibitors [41,42,43,44], this construct was used as a surrogate to study immature particle morphology.

The electron density of the HTLV-1 Gag lattice appears more compact than what has been previously observed for Gag lattices from HIV-1, MPMV or RSV [9,28,45]. The most intriguing morphological feature of the HTLV-1 immature Gag lattice is that about 20% of the HTLV-1-like particles had regions that appeared to be flat and did not follow the curvature of the viral membrane in multiple regions. The maximum separation between these ‘flat’ regions and the viral membrane was approximately 8 nm. This is the first time this observation has been made regarding the structure of an immature retroviral Gag lattice. One intriguing possibility is that this morphological feature is indicative of a more rigid lattice structure compared to that of other previously reported HIV-1 immature Gag lattice structures. The addition of a fluorophore tag on the carboxy terminus of the HTLV-1 Gag protein did affect the diameter of the VLPs (*i.e.*, average diameter of 110 nm without tag *versus* 75 nm with tag) as well as the Gag-Gag interactions, given the distinct morphological differences in the presence and absence of the fluorophore tag [21]. This is in contrast to that observed with HIV-1 Gag, where particles produced from a Gag-YFP expression construct did not influence particle size [19]. Taken together, these results imply distinct differences in the Gag assemblies in HTLV-1 immature particles compared to that of other retroviruses, particularly HIV-1.

STEM analysis led to the observation that the HTLV-1 Gag copy number distribution per particle spanned a wide range for both HTLV-1-like particles and HTLV-1 authentic particles (Figure 4B and Figure 5B), which corresponded to the diverse particle size population (Figure 1C and Figure 3B). HTLV-1-like particles and mature particles were found to have Gag copy numbers of 1300–1600 and 1500–1900 Gag molecules/particle, respectively, which is in the general range of Gag copy numbers observed for other retroviruses including MPMV and RSV [17,18].

The observations made by this study emphasize both unique and common morphological features of the HTLV-1-like particles in comparison to other retrovirus immature VLPs. Future studies will include a detailed determination of the immature Gag lattice, which should provide important new insights into the unique aspects of HTLV-1 particle assembly, in particular, and new insights into retroviral assembly, in general.

## Figures and Tables

**Table 1:** Summary of the mass determinations and the calculated Gag copy number per particle in human T-cell leukemia virus type 1 (HTLV-1)-like particles and authentic HTLV-1 particles.

		HTLV-1 Particle Sample	
Measurement		Virus-Like Particle	Authentic Particle
Average Diameter (nm) ^a^		110	113
Average Particle Mass (MDa) ^b^		174	204
Mass of RNA, Lipid and Protein (MDa)	RNA ^c^	7	7
	Lipid ^d^	70	80
	Total protein ^e^	97	118
Mass of Gag Molecules (MDa)	Total Gag polyprotein ^f^	70–87	82–106
	Gag	70–87	70–90
	Gag-Pro	N/A	10–13
	Gag-Pro-Pol	N/A	2.5–3
Gag polyprotein copy number ^g^		1300–1600	1500–1900

^a^ As determined by cryogenic transmission electron microscopy (Cryo-TEM); ^b^ As determined by scanning transmission electron microscopy (STEM); ^c^ Mass contributed by RNA in virus-like particles was estimated experimentally as described in the Materials and Methods. The RNA mass contribution for authentic HTLV-1 particles was estimated based upon the genome size; ^d^ Mass contributed by lipids was estimated from average particle size and membrane thickness; ^e^ Mass of total protein was determined by subtraction of the RNA and lipid mass from the total particle mass as determined by STEM; ^f^ Total Gag polyprotein was estimated based upon the assumption that Gag contributes ~70%–90% of the total protein mass; ^g^ The Gag polyprotein copy number represents the range of Gag copy number in a particle that has both average mass and dimensions.

## Abbreviations

The following abbreviations are used in this manuscript:

AT-2	2,2′-dithiodipyridine
BNL	Brookhaven National Laboratory
Cryo-TEM	cryogenic transmission electron microscopy
HIV-1	human immunodeficiency virus type 1
HTLV-1	human T-cell leukemia virus type 1
MPMV	Mason-Pfizer monkey virus
RSV	sarcoma virus
STEM	scanning transmission electron microscopy
TMV	tobacco mosaic virus
VLP	virus-like particle

## References

[1] De The G., Bomford R. (1993). An HTLV-1 vaccine: Why, how, for whom?. AIDS Res. Hum. Retrovir..

[2] Gessain A., Cassar O. (2012). Epidemiological aspects and world distribution of HTLV-1 infection. Front. Microbiol..

[3] Gessain A., Barin F., Vernant J.C., Gout O., Maurs L., Calender A., de The G. (1985). Antibodies to human T-lymphotropic virus type-1 in patients with tropical spastic paraparesis. Lancet.

[4] Osame M., Usuku K., Izumo S., Ijichi N., Amitani H., Igata A., Matsumoto M., Tara M. (1986). HTLV-1 associated myelopathy, a new clinical entity. Lancet.

[5] Maldonado J., Martin J., Mueller J., Zhang W., Mansky L. (2014). New insights into retroviral Gag-Gag and Gag-membrane interactions. Front. Microbiol..

[6] Fogarty K.H., Chen Y., Grigsby I.F., Macdonald P.J., Smith E.M., Johnson J.L., Rawson J.M., Mansky L.M., Mueller J.D. (2011). Characterization of cytoplasmic Gag-Gag interactions by dual-color z-scan fluorescence fluctuation spectroscopy. Biophys. J..

[7] Jouvenet N., Simon S.M., Bieniasz P.D. (2009). Imaging the interaction of HIV-1 genomes and Gag during assembly of individual viral particles. Proc. Natl. Acad. Sci. USA.

[8] Kingston R.L., Olson N.H., Vogt V.M. (2001). The organization of mature Rous sarcoma virus as studied by cryoelectron microscopy. J. Struct. Biol..

[9] Briggs J.A., Johnson M.C., Simon M.N., Fuller S.D., Vogt V.M. (2006). Cryo-electron microscopy reveals conserved and divergent features of Gag packing in immature particles of Rous sarcoma virus and human immunodeficiency virus. J. Mol. Biol..

[10] Briggs J.A., Simon M.N., Gross I., Krausslich H.G., Fuller S.D., Vogt V.M., Johnson M.C. (2004). The stoichiometry of Gag protein in HIV-1. Nat. Struct. Mol. Biol..

[11] Briggs J.A., Watson B.E., Gowen B.E., Fuller S.D. (2004). Cryoelectron microscopy of mouse mammary tumor virus. J. Virol..

[12] Yeager M., Wilson-Kubalek E.M., Weiner S.G., Brown P.O., Rein A. (1998). Supramolecular organization of immature and mature murine leukemia virus revealed by electron cryo-microscopy: Implications for retroviral assembly mechanisms. Proc. Natl. Acad. Sci. USA.

[13] Fuller S.D., Wilk T., Gowen B.E., Krausslich H.G., Vogt V.M. (1997). Cryo-electron microscopy reveals ordered domains in the immature HIV-1 particle. Curr. Biol..

[14] Butan C., Winkler D.C., Heymann J.B., Craven R.C., Steven A.C. (2008). RSV capsid polymorphism correlates with polymerization efficiency and envelope glycoprotein content: Implications that nucleation controls morphogenesis. J. Mol. Biol..

[15] Yu F., Joshi S.M., Ma Y.M., Kingston R.L., Simon M.N., Vogt V.M. (2001). Characterization of Rous sarcoma virus Gag particles assembled *in vitro*. J. Virol..

[16] Carlson L.A., Briggs J.A., Glass B., Riches J.D., Simon M.N., Johnson M.C., Muller B., Grunewald K., Krausslich H.G. (2008). Three-dimensional analysis of budding sites and released virus suggests a revised model for HIV-1 morphogenesis. Cell Host Microbe.

[17] Vogt V.M., Simon M.N. (1999). Mass determination of Rous sarcoma virus virions by scanning transmission electron microscopy. J. Virol..

[18] Parker S.D., Wall J.S., Hunter E. (2001). Analysis of Mason-Pfizer monkey virus Gag particles by scanning transmission electron microscopy. J. Virol..

[19] Chen Y., Wu B., Musier-Forsyth K., Mansky L.M., Mueller J.D. (2009). Fluorescence fluctuation spectroscopy on viral-like particles reveals variable gag stoichiometry. Biophys. J..

[20] Zhang W., Cao S., Martin J.L., Mueller J.D., Mansky L.M. (2015). Morphology and ultrastructure of retrovirus particles. AIMS Biophys..

[21] Grigsby I.F., Zhang W., Johnson J.L., Fogarty K.H., Chen Y., Rawson J.M., Crosby A.J., Mueller J.D., Mansky L.M. (2010). Biophysical analysis of HTLV-1 particles reveals novel insights into particle morphology and Gag stochiometry. Retrovirology.

[22] Haertle T., Carrera C.J., Wasson D.B., Sowers L.C., Richman D.D., Carson D.A. (1988). Metabolism and anti-human immunodeficiency virus-1 activity of 2-halo-2′, 3′-dideoxyadenosine derivatives. J. Biol. Chem..

[23] Harada S., Koyanagi Y., Yamamoto N. (1985). Infection of HTLV-III/LAV in HTLV-I-carrying cells MT-2 and MT-4 and application in a plaque assay. Science.

[24] Rossio J.L., Esser M.T., Suryanarayana K., Schneider D.K., Bess J.W., Vasquez G.M., Wiltrout T.A., Chertova E., Grimes M.K., Sattentau Q. (1998). Inactivation of human immunodeficiency virus type 1 infectivity with preservation of conformational and functional integrity of virion surface proteins. J. Virol..

[25] Baker T.S., Olson N.H., Fuller S.D. (1999). Adding the third dimension to virus life cycles: Three-dimensional reconstruction of icosahedral viruses from cryo-electron micrographs. Microbiol. Mol. Biol. Rev..

[26] Wall J.S., Hainfeld J.F., Simon M.N. (1998). Scanning transmission electron microscopy of nuclear structures. Methods Cell Biol..

[27] Namba K., Stubbs G. (1986). Structure of tobacco mosaic virus at 3.6 Å resolution: Implications for assembly. Science.

[28] Bharat T.A., Davey N.E., Ulbrich P., Riches J.D., de Marco A., Rumlova M., Sachse C., Ruml T., Briggs J.A. (2012). Structure of the immature retroviral capsid at 8 Å resolution by cryo-electron microscopy. Nature.

[29] de Marco A., Davey N.E., Ulbrich P., Phillips J.M., Lux V., Riches J.D., Fuzik T., Ruml T., Krausslich H.G., Vogt V.M. (2010). Conserved and variable features of Gag structure and arrangement in immature retrovirus particles. J. Virol..

[30] Frank G.A., Narayan K., Bess J.W., Del Prete G.Q., Wu X., Moran A., Hartnell L.M., Earl L.A., Lifson J.D., Subramaniam S. (2015). Maturation of the HIV-1 core by a non-diffusional phase transition. Nat. Commun..

[31] Briggs J.A., Riches J.D., Glass B., Bartonova V., Zanetti G., Krausslich H.G. (2009). Structure and assembly of immature HIV. Proc. Natl. Acad. Sci. USA.

[32] Wright E.R., Schooler J.B., Ding H.J., Kieffer C., Fillmore C., Sundquist W.I., Jensen G.J. (2007). Electron cryotomography of immature HIV-1 virions reveals the structure of the CA and SP1 Gag shells. EMBO J..

[33] Cao S., Maldonado J.O., Grigsby I.F., Mansky L.M., Zhang W. (2015). Analysis of human T-cell leukemia virus type 1 particles by using cryo-electron tomography. J. Virol..

[34] Cockburn J.J., Abrescia N.G., Grimes J.M., Sutton G.C., Diprose J.M., Benevides J.M., Thomas G.J., Bamford J.K., Bamford D.H., Stuart D.I. (2004). Membrane structure and interactions with protein and DNA in bacteriophage PRD1. Nature.

[35] Fleissner E. (1971). Chromatographic separation and antigenic analysis of proteins of the oncornaviruses. I. Avian leukemia-sarcoma viruses. J. Virol..

[36] Nam S.H., Kidokoro M., Shida H., Hatanaka M. (1988). Processing of Gag precursor polyprotein of human T-cell leukemia virus type I by virus-encoded protease. J. Virol..

[37] Nam S.H., Copeland T.D., Hatanaka M., Oroszlan S. (1993). Characterization of ribosomal frameshifting for expression of pol gene products of human T-cell leukemia virus type I. J. Virol..

[38] Mador N., Panet A., Honigman A. (1989). Translation of gag, pro, and pol gene products of human T-cell leukemia virus type 2. J. Virol..

[39] Poiesz B.J., Ruscetti F.W., Gazdar A.F., Bunn P.A., Minna J.D., Gallo R.C. (1980). Detection and isolation of type C retrovirus particles from fresh and cultured lymphocytes of a patient with cutaneous T-cell lymphoma. Proc. Natl. Acad. Sci. USA.

[40] Poiesz B.J., Ruscetti F.W., Reitz M.S., Kalyanaraman V.S., Gallo R.C. (1981). Isolation of a new type C retrovirus (HTLV) in primary uncultured cells of a patient with sezary T-cell leukaemia. Nature.

[41] Ding Y.S., Rich D.H., Ikeda R.A. (1998). Substrates and inhibitors of human T-cell leukemia virus type I protease. Biochemistry.

[42] Pettit S.C., Sanchez R., Smith T., Wehbie R., Derse D., Swanstrom R. (1998). HIV type 1 protease inhibitors fail to inhibit HTLV-I Gag processing in infected cells. AIDS Res. Hum. Retrovir..

[43] Louis J.M., Oroszlan S., Tozser J. (1999). Stabilization from autoproteolysis and kinetic characterization of the human T-cell leukemia virus type 1 proteinase. J. Biol. Chem..

[44] Tozser J., Weber I.T. (2007). The protease of human T-cell leukemia virus type-1 is a potential therapeutic target. Curr. Pharm. Des..

[45] Schur F.K., Hagen W.J., Rumlova M., Ruml T., Muller B., Krausslich H.G., Briggs J.A. (2015). Structure of the immature HIV-1 capsid in intact virus particles at 8.8 Å resolution. Nature.

